# Role of Phosphorus-Containing Molecules on the Formation of Nano-Sized Calcium Phosphate for Bone Therapy

**DOI:** 10.3389/fbioe.2022.875531

**Published:** 2022-06-22

**Authors:** Yingying Jiang, Yali Tao, Yutong Chen, Xu Xue, Gangyi Ding, Sicheng Wang, Guodong Liu, Mengmeng Li, Jiacan Su

**Affiliations:** ^1^ Institute of Translational Medicine, Shanghai University, Shanghai, China; ^2^ Department of Orthopedic, Spinal Pain Research Institute, Shanghai Tenth People’s Hospital, Tongji University School of Medicine, Shanghai, China; ^3^ Department of Orthopedics Trauma, Shanghai Zhongye Hospital, Shanghai, China; ^4^ Wound Care Center, Daping Hospital, Army Medical Center of PLA, Chongqing, China; ^5^ Department of Trauma Orthopedics, Changhai Hospital, Naval Medical University, Shanghai, China

**Keywords:** phosphorus-containing molecules, calcium phosphate, nanocomposites, bone therapy, drug delivery

## Abstract

Calcium phosphate (CaP) is the principal inorganic constituent of bone and teeth in vertebrates and has various applications in biomedical areas. Among various types of CaPs, amorphous calcium phosphate (ACP) is considered to have superior bioactivity and biodegradability. With regard to the instability of ACP, the phosphorus-containing molecules are usually adopted to solve this issue, but the specific roles of the molecules in the formation of nano-sized CaP have not been clearly clarified yet. Herein, alendronate, cyclophosphamide, zoledronate, and foscarnet are selected as the model molecules, and theoretical calculations were performed to elucidate the interaction between calcium ions and different model molecules. Subsequently, CaPs were prepared with the addition of the phosphorus-containing molecules. It is found that cyclophosphamide has limited influence on the generation of CaPs due to their weak interaction. During the co-precipitation process of Ca^2+^ and PO_4_^3-^, the competitive relation among alendronate, zoledronate, and foscarnet plays critical roles in the produced inorganic-organic complex. Moreover, the biocompatibility of CaPs was also systematically evaluated. The DFT calculation provides a convincing strategy for predicting the structure of CaPs with various additives. This work is promising for designing CaP-based multifunctional drug delivery systems and tissue engineering materials.

## Introduction

Bones are indispensable tissues in the body and perform plenty of vital functions, such as facilitating locomotion, maintaining the balance of calcium and phosphate, harboring the bone marrow, and protecting organs as well as supporting soft tissues (Grabowski, 2015). Bones make great contributions toward good health, but various bone diseases (Jiang et al., 2022), including scoliosis, fractures (Kellam et al., 2018), osteoporosis (Li et al., 2020), osteoarthritis (Hu et al., 2021), and bone cancer (Xue et al., 2021), are extremely disruptive to people’s quality of life.

Calcium phosphate (CaP) is the principal inorganic constituent of bones and teeth in vertebrates (Dorozhkin and Epple, 2002; Sawamoto et al., 2020). The CaP materials are essential biomaterials with excellent biocompatibility, and therefore, they have wide applications in biomedical areas, including bone regeneration and coating of implants or fillers in bones or teeth (Wang and Yeung, 2017; Li et al., 2021). Compared to normal non-degradable drug carriers, CaP materials exhibit superior advantages such as excellent biodegradability and biological responses, since the metabolites are ubiquitous ions of calcium and phosphate (Pina et al., 2015; Jiang et al., 2018).

Among the multifarious types of calcium phosphate, amorphous calcium phosphate (ACP) is the primary phase that can first precipitate from super-saturated aqueous solution owing to its low surface energy relative to that of hydroxyapatite (HAP) and octacalcium phosphate (OCP) (Sawamoto et al., 2020). Moreover, ACP is a relatively outstanding biomaterial with superior biodegradability and osteoinductivity (Qi et al., 2019). While ACP is quite unstable in aqueous solution, it will transform into crystalline HAP in a short time (Kwak et al., 2014). It should be noted that the metastable ACP phase can be stabilized well in aqueous solution using appropriate additives (Manuel Delgado-Lopez et al., 2017; Mao et al., 2021; Ruiz-Agudo et al., 2021) and/or ions (Ding et al., 2014). There are numerous excellent previous studies focusing on the regulation of calcium phosphate with various morphologies, such as by adding polymers (Yao et al., 2019), citric acid (von Schirnding et al., 2021), oleic acid (Li et al., 2017), and nucleic acid (Shen et al., 2021). It has been demonstrated that the formation of calcium phosphate can be influenced by the polarity, molecular weight, and electric groups of polymers (Schweizer and Taubert, 2007). Citric acids can be bonded to the surface of calcium phosphate to suppress the occurrence of crystal nucleation, and thereby act as inhibitors of HAP crystallization (Johnsson et al., 1991). The carboxyl group of citric acid competes with the phosphate group to bind with free calcium ions, thus stabilizing amorphous calcium phosphate (Ruiz-Agudo et al., 2021). Calcium oleate can be used as the precursor to tune the hydrophilicity/hydrophobicity, and then ultralong HAP nanowires with high flexibility can be achieved.

In addition, it has been commonly recognized that phosphorus-containing molecules (Fleisch, 1998; Zhou et al., 2020) can regulate the formation and growth of inorganic nanocrystals. Phosphorus-containing molecules, such as bisphosphonates, which have been widely used for clinical treatment, can work as additives to synthesize CaPs due to their strong affinity with calcium (Wang et al., 2015; Li et al., 2016). However, the specific role of phosphorus-containing molecules in the formation of nano-sized CaPs has not been clarified yet.

Herein, several phosphorus-containing molecules were selected to explore the mechanisms. Firstly, a density functional theory (DFT) calculation was conducted to predict the interaction between calcium ions and molecules. Afterwards, the phosphorus-containing molecules were incorporated to prepare CaPs, and the morphology, structure evolution, and cell viability of the synthesized materials were systematically characterized and evaluated. The experimental results were consistent with the DFT calculation, indicating that strong interactions between calcium ions and phosphorus-containing molecules are essential for the regulation of the growth and morphology of CaP nanocrystals. Furthermore, the DFT calculation can also provide guidance for the design and preparation of CaPs with various functions. Accordingly, various CaP nano-materials are promising for applications in multifunctional drug delivery systems and tissue engineering scaffolds.

## Experiment

### Materials

All the chemicals used for the experiments were of analytical grade, and they were directly used as received without any further purification. CaCl_2_, NaOH, KH_2_PO_4_, Na_2_HPO_4_, NaCl, KCl, alendronate sodium (C_4_H_12_NaNO_7_P_2_·3H_2_O, Adn), cyclophosphamide (C_7_H_15_C_l2_N_2_O_2_P, Cpp), zoledronic acid monohydrate (C_5_H_10_N_2_O_7_P_2_·H_2_O, Zda), and foscarnet sodium (CNa_3_O_5_P, Fss) were purchased from Aladdin Industrial Co., Ltd.; 2× phosphate-buffered saline (2× PBS) was prepared by successively dissolving NaCl, KCl, Na2HPO4, and KH2PO_4_ in deionized water with the concentrations of 272 mM, 5.2 mM, 16 mM, and 4 mM, respectively. The final solution has a pH value of about 7.4 at room temperature.

### Density Functional Theory Calculation

Theoretical calculations were performed employing the ORCA program (Neese et al., 2009) using a density functional theory (DFT)–based methodology. The geometries of all the structures were optimized with the hybrid B3LYP functional and the 6-31G (d) basis set to rationalize the interaction. Accordingly, the Gibbs free energies were further obtained with the hybrid B3LYP functional and the 6-311G (d, p) basis set.

### Preparation of CaP With the Incorporation of Phosphorus-Containing Molecules

In a typical synthesis procedure, 20 ml 2 × PBS solution was added to 20 ml of a solution containing CaCl_2_ and phosphorus-containing molecules with a concentration of 33.4 mM, NaOH (0.2 M) was introduced to adjust the pH of the mixed solution to 7.4, and then the mixture was placed in a water bath at 37°C and subjected to magnetic stirring for 1 h. The products were collected *via* centrifugation and washed with deionized water and ethanol three times, and then freeze-dried for further characterization. Reactions were also conducted without the addition of phosphorus-containing molecules, and the products were denoted as Control CaPs. The CaP samples prepared under the regulation of Adn, Cpp, Zda, and Fss were marked as CaP/Adn, CaP/Cpp, CaP/Zda, and CaP/Fss, respectively. Moreover, the co-precipitation products formed at 10 min were also collected and denoted as CaP/And-0, CaP/Cpp-0, CaP/Zda-0, and CaP/Fss-0, respectively. Thereafter, Adn or Zda with a concentration of 3.34 mM in the mixed solution was also conducted to obtain CaP products with different drug contents, and the samples were marked as CaP/Adn-1 and CaP/Zda-1.

### Loading Capacity and Releasing Profile of the Four Molecules

The dried powders (5 mg) of the as-prepared CaP/Cpp, CaP/Zda, and CaP/Fss were dispersed in 5 ml of PBS solution (pH 7.4) to reveal the release kinetics of the incorporated molecules from the corresponding CaP products. The suspensions were placed in a shaker with a constant shaking frequency of 140 rpm at 37°C. The supernatants and sediments were collected at given time points of 1, 6, 12, 24, and 48 h, respectively. The UV-Vis absorption curves of Cpp, Zda, and Fss with different concentrations were measured, and the related curves are shown in Supplementary Figures S5A,B, S6A. The UV-Vis absorption curves of the released medium at different time points were also recorded. Moreover, to detect the incorporated molecules in CaP samples, 0.25 mg of CaP/Cpp, CaP/Zda, CaP/Zda-1, and 1 mg of CaP/Fss were dissolved in 1 ml of HCl (1M), and the UV-Vis absorption curves were also recorded and shown in Supplementary Figures S5C,D, S6D.

### Structural and Chemical Characterization

The X-ray powder diffraction (XRD) patterns of the CaP products were carried out using an X-ray powder diffractometer (Bruker advance D8, Germany) supplemented with Rigaku D/max 40 kV and Cu Kα radiation. Fourier transform infrared (FTIR) spectra were collected *via* an FTIR spectrometer (Nicolet iS5, Thermo Scientific, USA). Transmission electron microscopy (TEM) micrographs of the as-prepared CaP products were taken with a field-emission electron microscope (JEOL JEM-2100F, Japan) associated with energy-dispersive spectroscopy (EDS, JED2300).

### Cell Viability Tests *in vitro*

Mouse bone marrow–derived mesenchymal stem cells (BMSCs) were purchased from Cyagen Biosciences Incorporation (China) and cultured in a low-glucose Dulbecco’s minimum essential medium (DMEM (LG), Sigma Life Science), supplemented with 10% fetal bovine serum (FBS) and 1% penicillin–streptomycin (PS). Sprague–Dawley rat–derived osteoblast-like UMR106 cells were obtained from the Cell Resources Center, Chinese Academy of Sciences (Shanghai), and cultured in a high-glucose DMEM (Sigma Life Science) containing 10% FBS and 1% PS. The cell incubator was set at 37°C and 5% CO_2_ following the manufacturer’s instructions. BMSCs from passage 3 to 5 were used for further experiments.

BMSCs and UMR-106s were seeded in the 96-well plate with a density of 2,000 cells per well. After being sterilized under ultraviolet light for 30 min, CaP products were co-cultured with BMSCs and UMR-106s with different concentrations. CCK8 assay was applied for testing the biocompatibility and cancer-killing effects of the CaP nanocomposites. After incubation for 2 days separately, the cell viability was assessed by CCK8 assay kits (DOJINDO, Japan). The detailed values were recorded *via* a microplate reader (BioTek Instruments, United States) at a wavelength of 450 nm. The corresponding results documented here were presented as an average value of at least four parallel measurements. Data were analyzed by GraphPad Prism 8.0 software, and comparisons were evaluated by Student’s t -test and ANOVA. Only when the *p* value < 0.05, the results were regarded as statistical significance.

## Results and Discussion

### Theoretical Simulation

**FIGURE 1:**
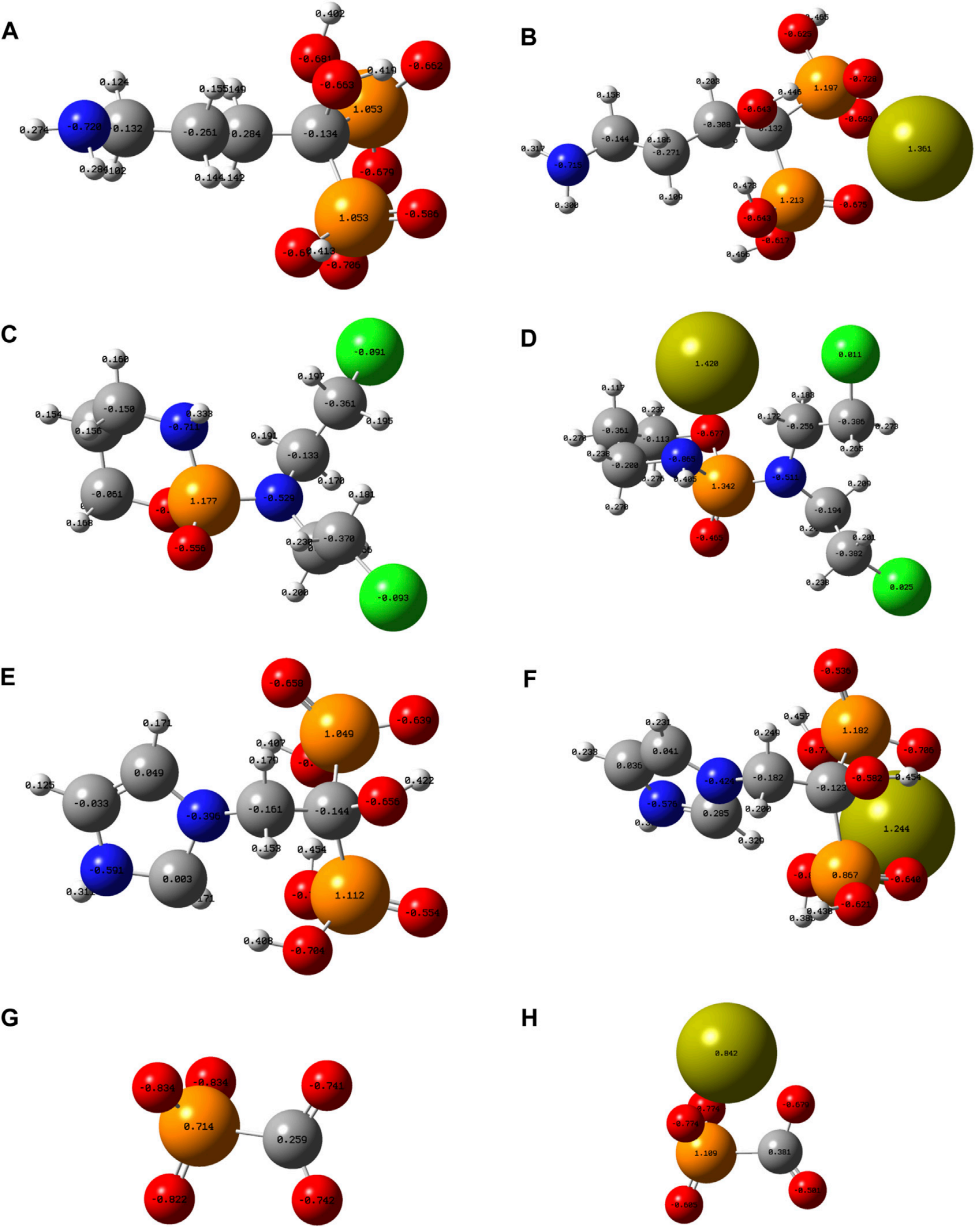
Optimized atomic structure showing the electrostatic density of **(A)** Adn^−^, **(B)** Adn^−^ + Ca^2+^, **(C)** Cpp, **(D)** Cpp + Ca^2+^, **(E)** Zda^-^, **(F)** Zda^−^ + Ca^2+^, **(G)** Fss^3-^, and **(H)** Fss^3-^ + Ca^2+^. Note: grey ball, carbon atom; white ball, hydrogen atom; blue ball, nitrogen atom; orange ball, phosphorus atom; lime-green ball, calcium atom; red ball, oxygen atom; and green ball, chlorine atom.

To theoretically reveal the role of phosphorus-containing molecules in the formation of CaPs, theoretical calculations were conducted employing the ORCA program (Neese et al., 2009) with the density functional theory (DFT) methodology at the B3LYP/6-31G (d) level. The optimized atomic structure and electrostatic density of phosphorus-containing molecules before and after the combination with calcium ions are shown in Figures 1A–H; the ionization state of each molecule at the pH of 7.4 was determined by their pKa values.The electrostatic density of certain oxygen atoms of the phosphate group becomes notably less positive after the combination with calcium ions, which indicates that the phosphate group and carboxyl group interact with calcium ions *via* the linkage of the Ca-O bond.

**FIGURE 2:**
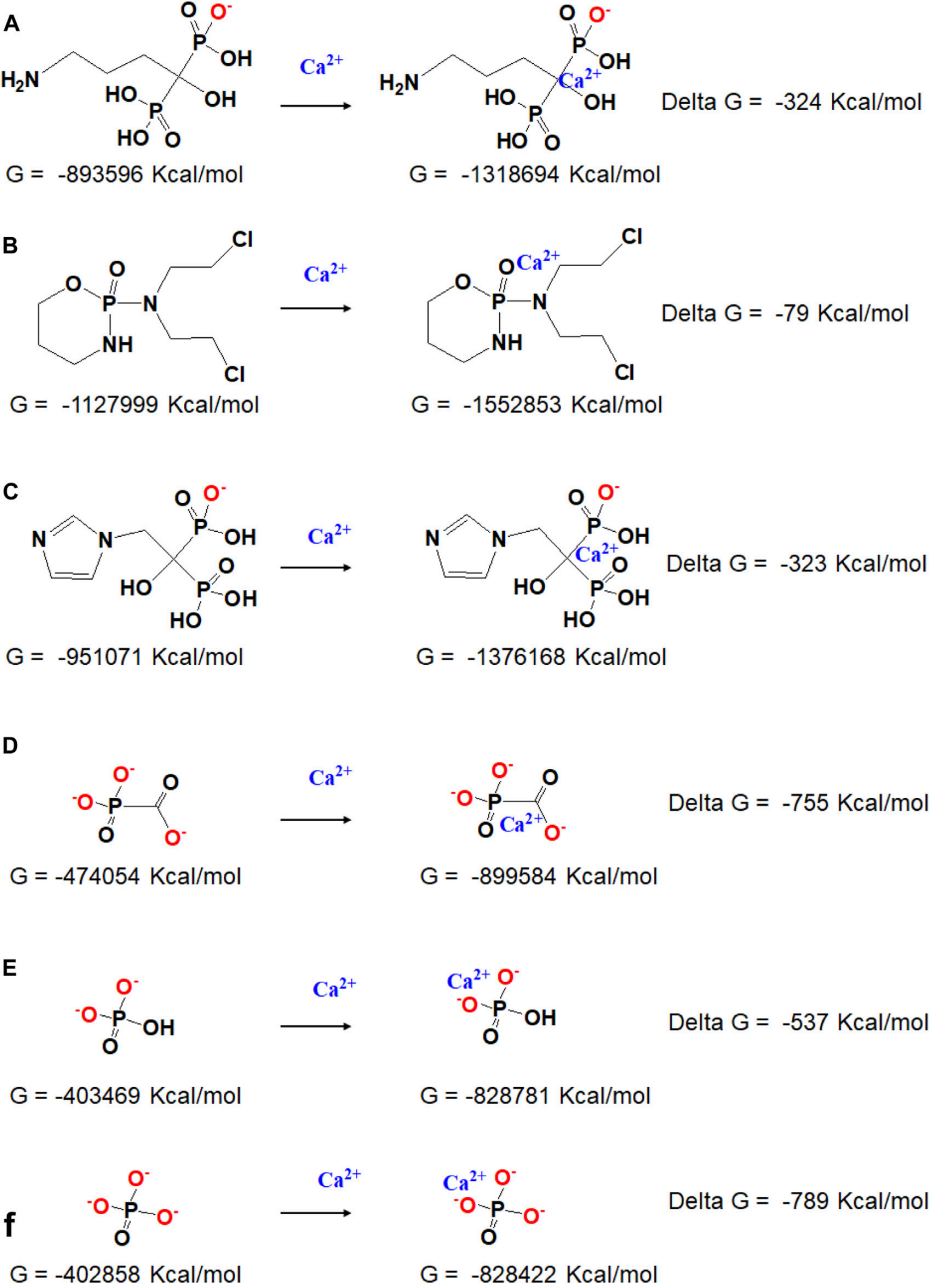
Chemical structural formula and the responding Gibbs free energy of **(A)** Adn^−^ and Adn^−^ + Ca^2+^, **(B)** Cpp and Cpp + Ca^2+^, **(C)** Zda^-^ and Zda^−^ + Ca^2+^, **(D)** Fss^3-^ and Fss^3-^ + Ca^2+^, **(E)** HPO_4_^2-^ and HPO_4_^2-^ + Ca^2+^, and **(F)** PO_4_^3-^ and PO_4_^3-^ + Ca^2+^.

Gibbs free energies (G) were further obtained with the hybrid B3LYP functional at the 6-311G (d, p) basis sets to quantificationally illustrate the interaction, and the detailed data under each chemical structural formula are also shown in Figure 2. The value of delta G represents the binding energy of phosphorus-containing molecules and calcium ions. It can be seen that the Cpp has the weakest interaction with Ca^2+^, Fss^3+^ combines closest with Ca^2+^, and the binding energy of Adn^−^ and Ca^2+^ is close to that of Zda^−^. According to the simulation results, the binding energy is probably related to the ionization state, charged group, and spatial configuration of the phosphorus-containing molecules.

It is reported that the phosphorus-containing molecules compete with the phosphate ions to react with the calcium ions (Fleisch, 1998), the simulation result also provided quantitative data about how strong the interaction is between each phosphorus-containing molecules and calcium ions. For Cpp, it has the weakest interaction with calcium, and the obtained CaP/Cpp has been rarely influenced by Cpp; Adn and Zda have a stronger binding affinity with calcium, while the binding energy is still lower than inorganic HPO_4_^2-^ and PO_4_^3-^ (Supplementary Figure S1); thereby, the crystal growth process of CaP is disturbed or some of the inorganic phosphates are replaced by Adn or Zda during the co-precipitation process, which leads to the formation of amorphous calcium phosphate or other new composites consisting of calcium and phosphorus-containing molecules; Fss and calcium have the strongest binding energy, which is higher than that of calcium and HPO_4_^2-^ but close to that of calcium and PO_4_^3-^ (Figures 2E,F). Considering the pH of the reaction system, HPO_4_^2-^ extensively exists in PBS (PH 7.4), Fss molecules may have an advantage over HPO_4_^2-^ to bind with calcium ions, and a new complex consisting of calcium and Fss could form.

### Synthesis and Characterization of CaPs With the Addition of Phosphorus-Containing Molecules

**FIGURE 3:**
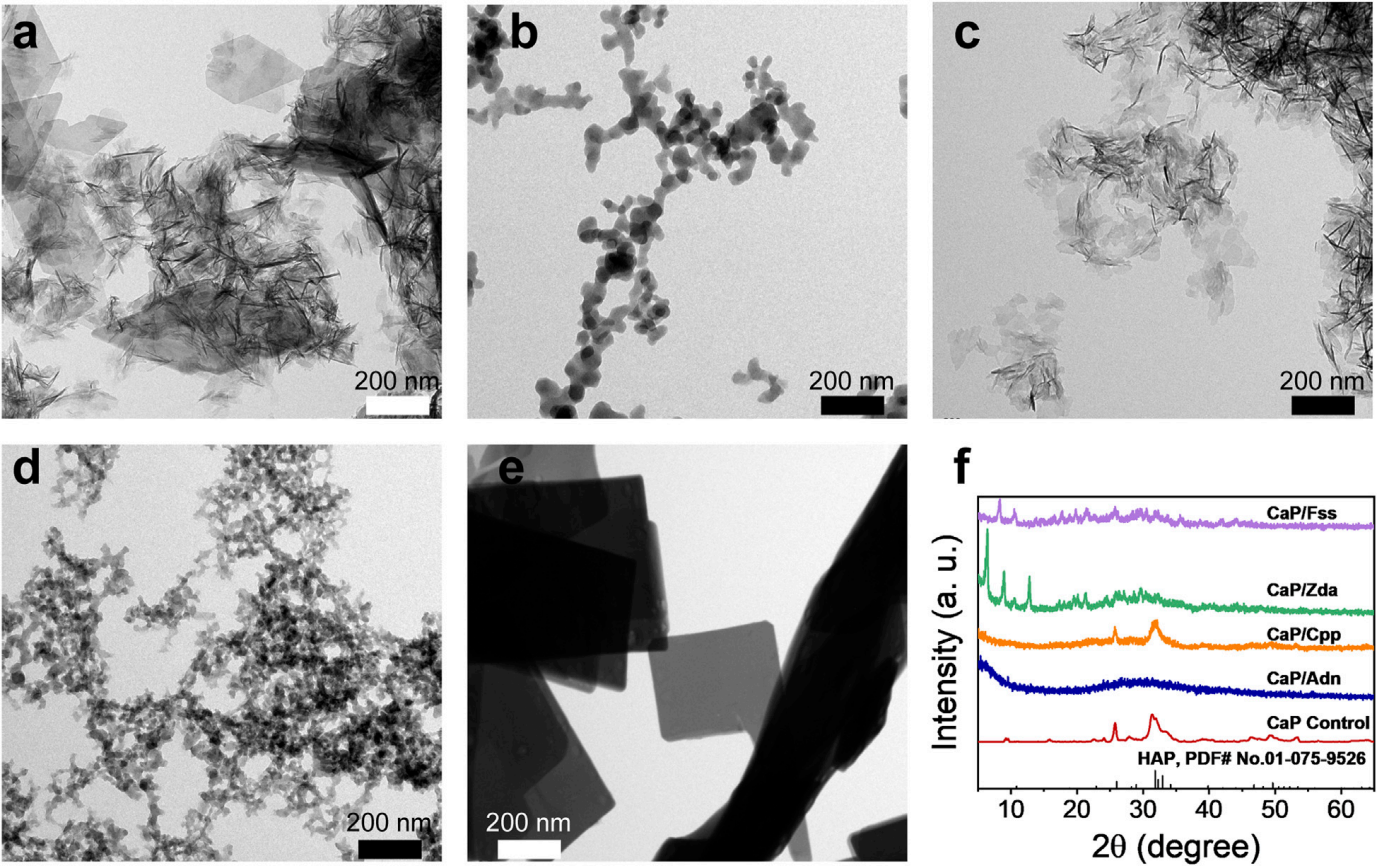
TEM micrographs of **(A)** CaP Control, **(B)** CaP/Adn, **(C)** CaP/Cpp, **(D)** CaP/Zda, and **(E)** CaP/Fss; **(F)** XRD patterns of CaP Control, CaP/Adn, CaP/Cpp, CaP/Zda, and CaP/Fss products.

To verify the results of the theoretical simulation, Adn^−^, Cpp, Zda^−^, and Fss^3-^ were selected as additives to regulate the formation of CaPs. Figure 3 provides the TEM micrographs of CaP Control prepared without adding phosphorus-containing molecules (Figure 3A), suggesting a nanosheet structure which is the typical morphology of CaP in bones. Both the CaP/Adn (Figure 3B) and CaP/Zda (Figure 3D) have a nanosized spheroidal structure with a diameter of 20–50 nm, whereas the grain size of CaP/Adn is obviously larger. Supplementary Figure S3 shows the TEM micrographs of CaP/Adn-0 and CaP/Zda-0, which also indicate the morphology of nanoparticles. It is found that Cpp has limited influence on the morphology of CaP/Cpp (Figure 3C). In contrast, CaP/Fss-0 (Supplementary Figure S3E) possesses a defective nanoplate structure, which is quite different from the other CaP products, but can be transformed into a perfect nanoplate with prolonged co-precipitation time (Figure 3E).

As the XRD patterns of CaP Control and CaP/Cpp given in Figure 3F show, their related characteristic diffraction peaks are located at 2θ of ∼32° but with weak intensities and large peak width, indicating a low-crystallinity apatite phase. CaP/Adn exhibits a typical amorphous structure, while CaP/Zda and CaP/Fss exhibited totally different features within the XRD patterns, which cannot be detected in the database of Joint Committee on Powder Diffraction Standards (JCPDS). As shown in Supplementary Figure S2, the XRD patterns of CaP/Zda matches those of calcium–Zda complexes extracted from cif data provided by Freire et al. (2010), indicating that CaP/Zda is a complex of calcium and Zda. According to the simulation results, Adn and Zda have close binding energy with calcium, while Adn decreased the crystallinity of CaP, which is also extensively reported (Huang et al., 2022), and Zda and calcium formed a complex with a higher crystallinity. CaP/Ada and CaP/Zda may have different atomic arrangements due to the different spatial configuration of Adn and Zda. The DFT simulation only calculated the binding energy of calcium and phosphorus-containing molecules, while the 3D arrangement of CaP products has not been taken into consideration, and the crystal structure may not be predicted *via* binding energy. Thereafter, FTIR spectra were collected to further investigate the chemical structure.

**FIGURE 4:**
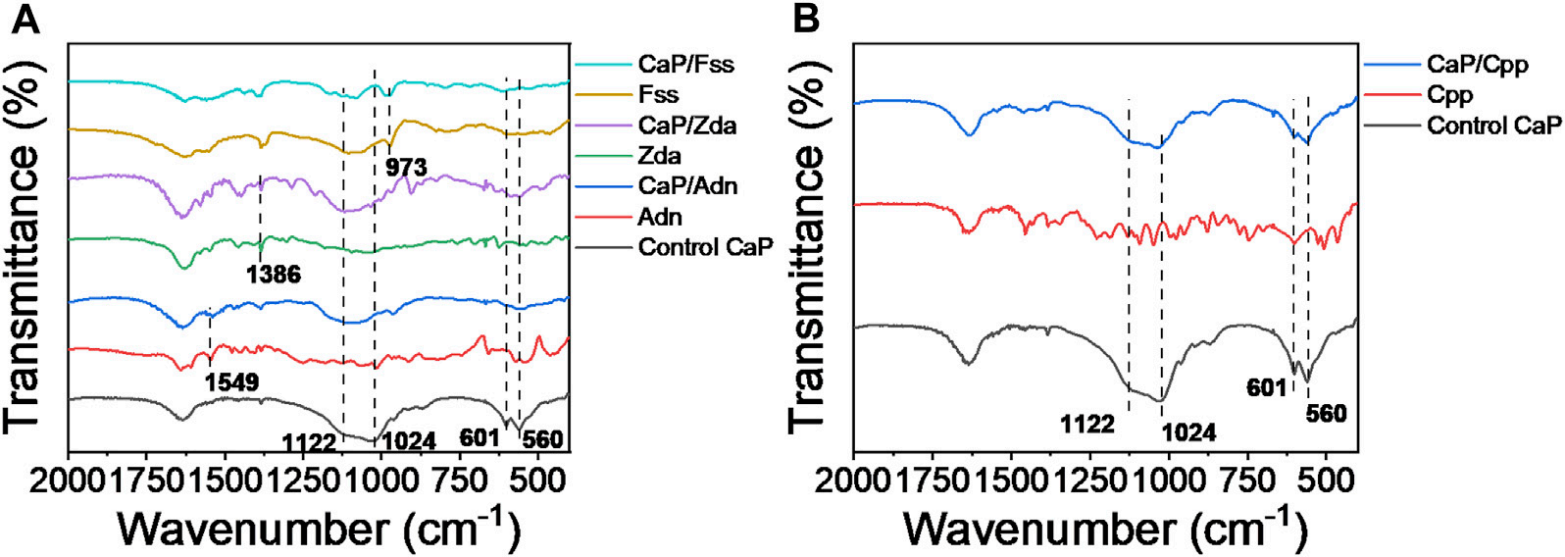
FTIR spectra of **(A)** CaP Control, Adn, CaP/Adn, Zda, CaP/Zda, Fss, and CaP/Fss; **(B)** CaP Control, Cpp, and CaP/Cpp.

As shown in Figure 4, the intense absorption peaks of CaP Control at about 1,122, 1,024, 601, and 560 cm^−1^ are attributed to the presence of the PO_3_^4-^ group. The typical features of Adn at 1,549 cm^−1^, Zda at 1,386 cm^−1^, and Fss at 973 cm^−1^ also appear on the spectra of CaP/Adn, CaP/Zda, and CaP/Fss, respectively, which reveals that the CaP products in Figure 4A are assigned to organic-inorganic complexes except for CaP Control. With comparisons, the typical features of Cpp are not obvious on the spectrum of CaP/Cpp, indicating the low content of Cpp and the weak interaction between Cpp and CaP. These experimental results are consistent with those calculated by a theoretical simulation.

**FIGURE 5:**
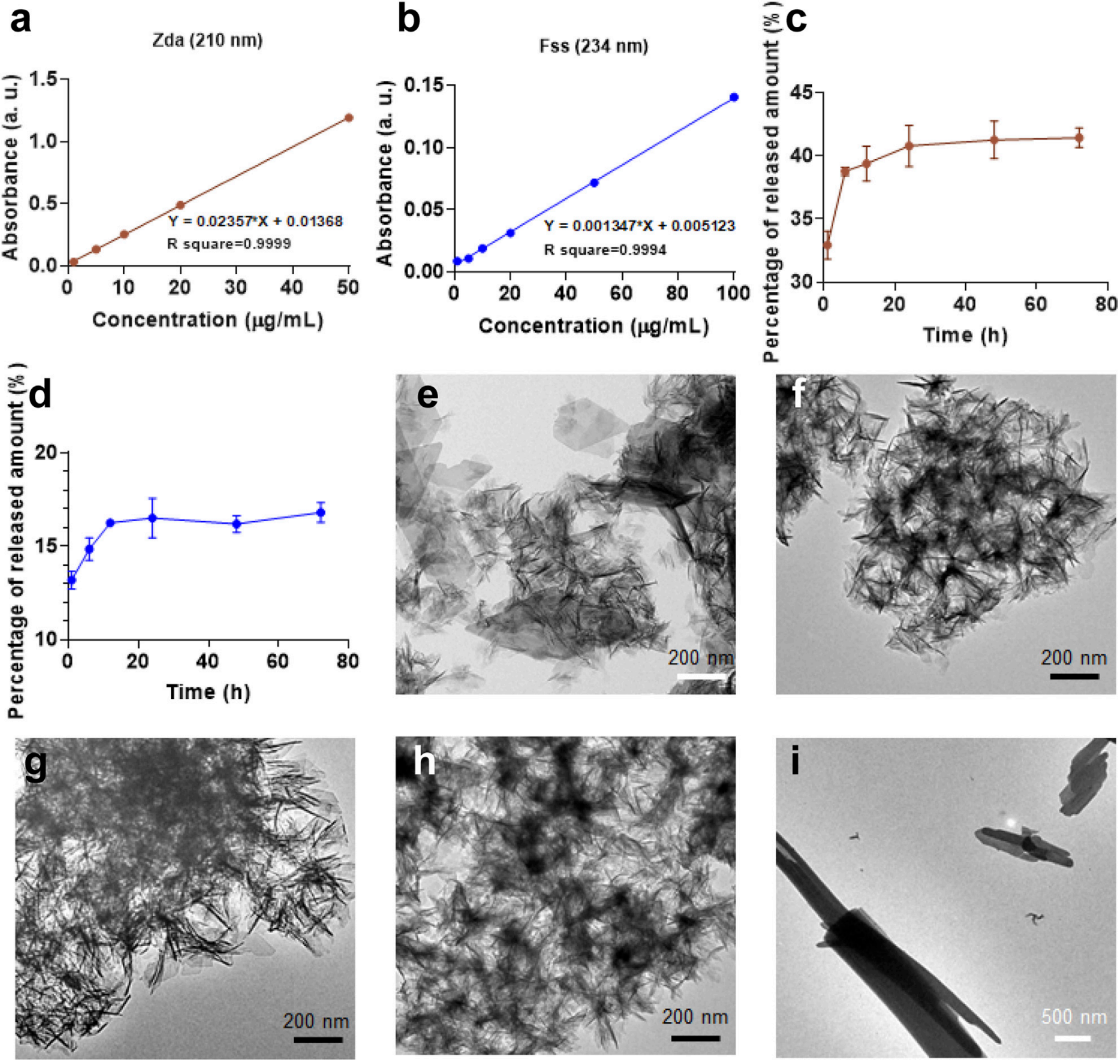
Absorbance-concentration curve of **(A)** Zda and **(B)** Fss obtained from related UV-Vis absorption curves in Supplementary Figure S5A,B; **(C)** Zda-release and **(D)** Fss-release curves of CaP/Zda and CaP/Fss in PBS. TEM micrographs of **(E)** CaP Control—72 h, **(F)** CaP/Adn—72 h, **(G)** CaP/Cpp—72 h, **(H)** CaP/Zda—72 h, and **(I)** CaP/Fss—72 h.

To further investigate the physicochemical property of the CaP products, loading capacity and drug-releasing profile of the molecules from the as-prepared CaP have been evaluated and shown in Figure 5 and Supplementary Figures S5, S6. In terms of the theoretical and experimental data, CaP/Adn and CaP/Zda are organic–inorganic complexes; meanwhile, Adn and Zda have similar binding energy with calcium, and the drug-release kinetics of CaP/Zda was examined due to the convenient detection. The R square of the absorbance-concentration curves of both Zda and Fss are above 0.999, indicating that an excellent linear fitting correlation has been obtained. Thereafter, the incorporated Zda and Fss in CaP/Zda, CaP/Zda-1, and CaP/Fss were tested and calculated; the corresponding drug-loading capacity are 175, 101, and 411 mg/g, respectively. Figures 5C,D show the release curves: the release of Zda and Fss reaches a plateau, or the release rate decreases after 24 h. CaP/Zda released about 40% of Zda after 24 h, while CaP/Fss only released 16% of Fss, suggesting a different transformation occurred in the system over 72 h. As shown in Figure 5H, after being shaken in PBS at 37°C for 72 h, the nanosized spherical CaP/Zda transformed into a similar nanosheet structure as CaP Control, indicating that Zda competes with the inorganic phosphate groups to react with calcium and the spherical morphology needs to be maintained by Zda. Meanwhile, CaP/Fss developed into a structure with a bunch of nanowires/nanorods (Figure 5I), and Fss which has a stronger binding affinity with calcium and played an essential role in the formation and transformation of CaP/Fss in the physiological environment. Moreover, Supplementary Figure S6 shows the UV-Vis absorption curves of the released medium of CaP and CaP/Cpp at different time points. Obviously, the UV-Vis absorption peak of Cpp is influenced by the dissolved CaP, and the peak intensity between dissolved CaP/Cpp and CaP are similar, revealing that the content of incorporated Cpp is quite low in CaP/Cpp. These results also explain the phenomenon that CaP/Cpp has a similar morphology to Cpp.

### Cytotoxicity Assay for the Biocompatibility of the CaPs

**FIGURE 6:**
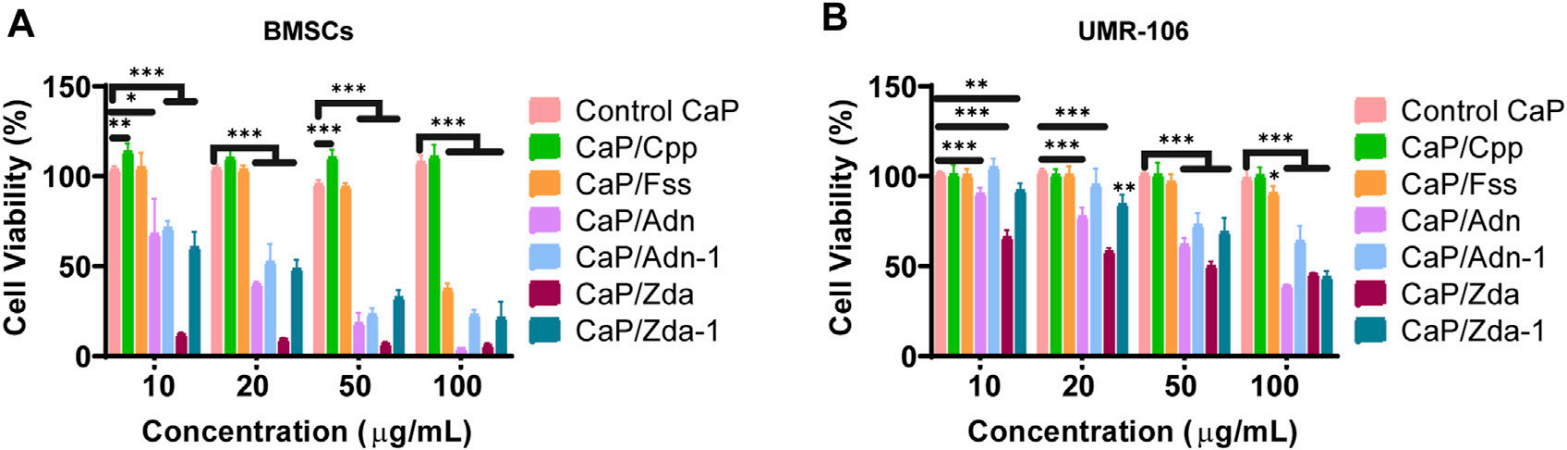
Cell viability of BMSCs **(A)** and UMR-106 **(B)** cells co-cultured with different CaPs at various concentrations for 48 h.

To evaluate the biocompatibility of CaPs with different structures, BMSCs and UMR-106 cells were co-cultured with the CaP products at a series of concentrations. As shown in Figure 6, CaP Control and CaP/Cpp exhibit excellent biocompatibility, and thus could promote the proliferation of BMSCs and UMR-106. CaP/Fss is non-toxic at a low concentration, while the biocompatibility is reduced when a concentration is set above 20 μg/ml. CaP/Adn and CaP/Zda show obvious cytotoxicity, and the cell viability is decreased with the concentration of nanoparticles increasing. The cytotoxicity of CaP/Adn and CaP/Zda is derived from the high content of Adn and Zda in the organic–inorganic complex. The nanocomplex can release phosphorus-containing drugs with degradation, as CaP is pH sensitive (Gou et al., 2016; Mi et al., 2016). Hence, Adn and Zda can realize controlled release under acid conditions(Huang et al., 2022). Since the bisphosphonates are reported to inactivate human epidermal growth factor receptors (human EGFR or HER) to perform antitumor actions (Yuen et al., 2014), CaP/Adn and CaP/Zda can be considered to be promising drug delivery systems for osteosarcoma therapy. CaP/Adn-1 (Supplementary Figure S4) and CaP/Zda-1 (Supplementary Figure S4B) loaded with fewer drugs showed better biocompatibility and are more suitable for biomedical uses.

## Conclusion

In this study, the interaction of calcium ions and phosphorus-containing molecules, including alendronate, cyclophosphamide, zoledronate, and foscarnet, were simulated *via* the ORCA program. It demonstrated that the electrostatic density of certain oxygen atoms in a phosphate group was less positive after the combination with calcium ions, indicating that the phosphate group and carboxyl group interacted with calcium ions *via* the linkage of a Ca-O bond. Meanwhile, the binding energy of calcium ions and each phosphorus-containing molecule were also obtained, and they were dependent upon the ionization state, charged group as well as spatial configuration. The theoretical simulation provided a prediction result of the role of phosphorus-containing molecules in the formation of nano-sized calcium phosphate. Subsequently, phosphorus-containing molecules were incorporated to prepare CaPs, and cyclophosphamide had limited influence on the formation of CaP due to their weak interaction. Adn, Zda, and Fss were competitive with the phosphate group during the coprecipitation process of Ca^2+^ and PO_4_^3-^, and played critical roles in the formation of the inorganic–organic complex. The experimental results were consistent with the DFT calculation, revealing that strong interactions between calcium ions and phosphorus-containing molecules were essential for the regulation of the growth and morphology of CaP nanocrystals. Additionally, the biocompatibility of CaPs was also evaluated, and cytotoxicity was mainly determined by the content and pharmacological property of the phosphorus-containing molecules. Overall, DFT calculation can provide a convincing strategy on predicating the structure of CaPs with various additives and the design of CaP-based multifunctional drug delivery systems and tissue engineering materials.

## Data Availability

The original contributions presented in the study are included in the article/Supplementary Material; further inquiries can be directed to the corresponding authors.

## References

[B1] Delgado-López J. M. Bertolotti F. Lyngsø J. Pedersen J. S. Cervellino A. Masciocchi N. (2017). The Synergic Role of Collagen and Citrate in Stabilizing Amorphous Calcium Phosphate Precursors with Platy Morphology. Acta Biomater. 49, 555–562. 10.1016/j.actbio.2016.11.041 27872013

[B2] Ding H. Pan H. Xu X. Tang R. (2014). Toward a Detailed Understanding of Magnesium Ions on Hydroxyapatite Crystallization Inhibition. Cryst. Growth & Des. 14 (2), 763–769. 10.1021/cg401619s

[B3] Dorozhkin S. V. Epple M. (2002). Biological and Medical Significance of Calcium Phosphates. Angew. Chem. Int. Ed. 41 (17), 3130–3146. 10.1002/1521-3773(20020902)41:17<3130::aid-anie3130>3.0.co;2-1 12207375

[B4] Fleisch H. (1998). Bisphosphonates: Mechanisms of Action. Endocr. Rev. 19 (1), 80–100. 10.1210/edrv.19.1.0325 9494781

[B5] Freire E. Vega D. R. Baggio R. (2010). Zoledronate Complexes. III. Two Zoledronate Complexes with Alkaline Earth Metals: [Mg(C5H9N2O7P2)2(H2O)2] and [Ca(C5H8N2O7P2)(H2O)]n. Acta Crystallogr. C 66, M166–M170. 10.1107/s0108270110017634 20522941

[B6] Gou M. Li S. Zhang L. Li L. Wang C. Su Z. (2016). Facile One-Pot Synthesis of Carbon/calcium phosphate/Fe3O4 Composite Nanoparticles for Simultaneous Imaging and pH/NIR-Responsive Drug Delivery. Chem. Commun. 52 (74), 11068–11071. 10.1039/c6cc05515j 27561158

[B7] Grabowski P. (2015). Physiology of Bone. Physiology Bone. Endocr. Dev. 28, 33–55. 10.1159/000380991 26138834

[B8] Hu Y. Chen X. Wang S. Jing Y. Su J. (2021). Subchondral Bone Microenvironment in Osteoarthritis and Pain. Bone Res. 9 (1), 20. 10.1038/s41413-021-00147-z 33731688 PMC7969608

[B9] Huang X. Qiu M. Wang T. Li B. Zhang S. Zhang T. (2022). Carrier-free Multifunctional Nanomedicine for Intraperitoneal Disseminated Ovarian Cancer Therapy. J. Nanobiotechnol. 20 (1), 93. 10.1186/s12951-022-01300-4 PMC8864853 35193583

[B10] Jiang Y.-Y. Zhou Z.-F. Zhu Y.-J. Chen F.-F. Lu B.-Q. Cao W.-T. (2018). Enzymatic Reaction Generates Biomimic Nanominerals with Superior Bioactivity. Small 14 (51), 1804321. 10.1002/smll.201804321 30417599

[B11] Jiang Y. Li J. Xue X. Yin Z. Xu K. Su J. (2022). Engineered Extracellular Vesicles for Bone Therapy. Nano Today 44, 101487. 10.1016/j.nantod.2022.101487

[B12] Johnsson M. Richardson C. F. Sallis J. D. Nancollas G. H. (1991). Adsorption and Mineralization Effects of Citrate and Phosphocitrate on Hydroxyapatite. Calcif. Tissue Int. 49 (2), 134–137. 10.1007/BF02565136 1655175

[B13] Kwak S.-Y. Kim S. Yamakoshi Y. Simmer J. P. Beniash E. Margolis H. C. (2014). Regulation of Calcium Phosphate Formation by Native Amelogenins *In Vitro* . Connect. Tissue Res. 55 (Suppl. 10), 21–24. 10.3109/03008207.2014.923853 25158174 PMC4145609

[B14] Li H. Zhu Y.-J. Jiang Y.-Y. Yu Y.-D. Chen F. Dong L.-Y. (2017). Hierarchical Assembly of Monodisperse Hydroxyapatite Nanowires and Construction of High-Strength Fire-Resistant Inorganic Paper with High-Temperature Flexibility. Chemnanomat 3 (4), 259–268. 10.1002/cnma.201700027

[B15] Li N. Song J. Zhu G. Shi X. Wang Y. (2016). Alendronate Conjugated Nanoparticles for Calcification Targeting. Colloids Surfaces B Biointerfaces 142, 344–350. 10.1016/j.colsurfb.2016.03.015 26970822

[B16] Li X. Wang L. Huang B. Gu Y. Luo Y. Zhi X. (2020). Targeting Actin-Bundling Protein L-Plastin as an Anabolic Therapy for Bone Loss. Sci. Adv. 6 (47), eabb7135. 10.1126/sciadv.abb7135 33208358 PMC7673802

[B17] Li Z. Du T. Ruan C. Niu X. (2021). Bioinspired Mineralized Collagen Scaffolds for Bone Tissue Engineering. Bioact. Mater. 6 (5), 1491–1511. 10.1016/j.bioactmat.2020.11.004 33294729 PMC7680706

[B18] Mao B. Xie Y. Yang H. Yu C. Ma P. You Z. (2021). Casein Phosphopeptide-Amorphous Calcium Phosphate Modified Glass Ionomer Cement Attenuates Demineralization and Modulates Biofilm Composition in Dental Caries. Dent. Mat. J. 40 (1), 84–93. 10.4012/dmj.2019-325 32908042

[B19] Meinberg E. G. Agel J. Roberts C. S. Karam M. D. Kellam J. F. Wilber J. H. (2018). Fracture and Dislocation Classification Compendium-2018. J. Orthop. Trauma 32 Suppl 1, S1–S170. 10.1097/BOT.0000000000001063 29256945

[B20] Mi P. Kokuryo D. Cabral H. Wu H. Terada Y. Saga T. (2016). A pH-Activatable Nanoparticle with Signal-Amplification Capabilities for Non-invasive Imaging of Tumour Malignancy. Nat. Nanotech 11 (8), 724–730. 10.1038/nnano.2016.72 27183055

[B21] Neese F. Wennmohs F. Hansen A. Becker U. (2009). Efficient, Approximate and Parallel Hartree-Fock and Hybrid DFT Calculations. A 'chain-Of-Spheres' Algorithm for the Hartree-Fock Exchange. Chem. Phys. 356 (1), 98–109. 10.1016/j.chemphys.2008.10.036

[B22] Pina S. Oliveira J. M. Reis R. L. (2015). Natural-Based Nanocomposites for Bone Tissue Engineering and Regenerative Medicine: A Review. Adv. Mat. 27 (7), 1143–1169. 10.1002/adma.201403354 25580589

[B23] Qi C. Musetti S. Fu L.-H. Zhu Y.-J. Huang L. (2019). Biomolecule-assisted Green Synthesis of Nanostructured Calcium Phosphates and Their Biomedical Applications. Chem. Soc. Rev. 48 (10), 2698–2737. 10.1039/c8cs00489g 31080987

[B24] Ruiz-Agudo E. Ruiz-Agudo C. Di Lorenzo F. Alvarez-Lloret P. Ibañez-Velasco A. Rodriguez-Navarro C. (2021). Citrate Stabilizes Hydroxylapatite Precursors: Implications for Bone Mineralization. ACS Biomater. Sci. Eng. 7 (6), 2346–2357. 10.1021/acsbiomaterials.1c00196 33973778 PMC8479724

[B25] Sawamoto K. Álvarez J. V. Herreño A. M. Otero-Espinar F. J. Couce M. L. Alméciga-Díaz C. J. (2020). Bone-Specific Drug Delivery for Osteoporosis and Rare Skeletal Disorders. Curr. Osteoporos. Rep. 18 (5), 515–525. 10.1007/s11914-020-00620-4 32845464 PMC7541793

[B26] Schweizer S. Taubert A. (2007). Polymer-controlled, Bio-Inspired Calcium Phosphate Mineralization from Aqueous Solution. Macromol. Biosci. 7 (9-10), 1085–1099. 10.1002/mabi.200600283 17712804

[B27] Shen M. j. Jiao K. Wang C. y. Ehrlich H. Wan M. c. Hao D. x. (2021). Extracellular DNA: A Missing Link in the Pathogenesis of Ectopic Mineralization. Adv. Sci. 9, 2103693. 10.1002/advs.202103693 PMC8844461 34939364

[B28] von Schirnding C. Giopanou I. Hermawan A. Wehl L. Ntaliarda G. Illes B. (2021). Synergistic Combination of Calcium and Citrate in Mesoporous Nanoparticles Targets Pleural Tumors. Chem 7 (2), 480–494. 10.1016/j.chempr.2020.11.021

[B29] Wang W. Yeung K. W. K. (2017). Bone Grafts and Biomaterials Substitutes for Bone Defect Repair: A Review. Bioact. Mater. 2 (4), 224–247. 10.1016/j.bioactmat.2017.05.007 29744432 PMC5935655

[B30] Wang Y. Zhu G. Li N. Song J. Wang L. Shi X. (2015). Small Molecules and Their Controlled Release that Induce the Osteogenic/chondrogenic Commitment of Stem Cells. Biotechnol. Adv. 33 (8), 1626–1640. 10.1016/j.biotechadv.2015.08.005 26341834

[B31] Xue X. Hu Y. Deng Y. Su J. (2021). Recent Advances in Design of Functional Biocompatible Hydrogels for Bone Tissue Engineering. Adv. Funct. Mat. 31 (19), 2009432. 10.1002/adfm.202009432

[B32] Yao S. Lin X. Xu Y. Chen Y. Qiu P. Shao C. (2019). Osteoporotic Bone Recovery by a Highly Bone‐Inductive Calcium Phosphate Polymer‐Induced Liquid‐Precursor. Adv. Sci. 6 (19), 1900683. 10.1002/advs.201900683 PMC6774089 31592093

[B33] Yuen T. Stachnik A. Iqbal J. Sgobba M. Gupta Y. Lu P. (2014). Bisphosphonates Inactivate Human EGFRs to Exert Antitumor Actions. Proc. Natl. Acad. Sci. U.S.A. 111 (50), 17989–17994. 10.1073/pnas.1421410111 25453081 PMC4273397

[B34] Zhou H. Jiang Y.-Y. Tan S. Liu L.-J. Yao Q.-T. Xia Y.-J. (2020). Flower-like Calcium Phosphoserine Complex as Biomimetic Mineral with High Bioactivity. Ceram. Int. 46 (13), 20914–20922. 10.1016/j.ceramint.2020.05.142

