# Supplementary material for: Role of Phosphorus-Containing Molecules on the Formation of Nano-Sized Calcium Phosphate for Bone Therapy
Source: Front Bioeng Biotechnol. 2022 Jun 22;10:875531. doi: 10.3389/fbioe.2022.875531 (PMC9257216; doi:10.3389/fbioe.2022.875531)
Supplement: Supplementary file 1 [file DataSheet1.pdf]

Role of phosphorus-containing molecules on the formation of nano-sized calcium phosphate for bone therapy

Yingying Jiang, [a, b, †] Yali Tao, [a, †] Yutong Chen, [a, †] Xu Xue, [a] Gangyi Ding, [a] Sicheng Wang, [a, c] Guodong Liu, [d, *] Mengmeng Li, [a, *] Jiacan Su [a, e, *]

a Institute of Translational Medicine, Shanghai University, Shanghai, 200444, P. R. China.
b, Department of Orthopedic, Spinal Pain Research Institute, Shanghai Tenth People's Hospital, Tongji University School of Medicine, Shanghai 200072, P. R. China,
c, Department of Orthopedics Trauma, Shanghai Zhongye Hospital, Shanghai, 200941, P. R. China
d, Wound Care Center, Daping Hospital, Army Medical Center of PLA, Chongqing 400042, P. R. China
e, Department of Trauma Orthopedics, Changhai Hospital, Naval Medical University, Shanghai, 200433, P. R. China

[†] Y. Jiang, Y. Tao and Y. Chen contributed equally.
* Corresponding author. E-mail: drsujiacan@163.com, mengmengli@shu.edu.cn, frankliugd@163.com.

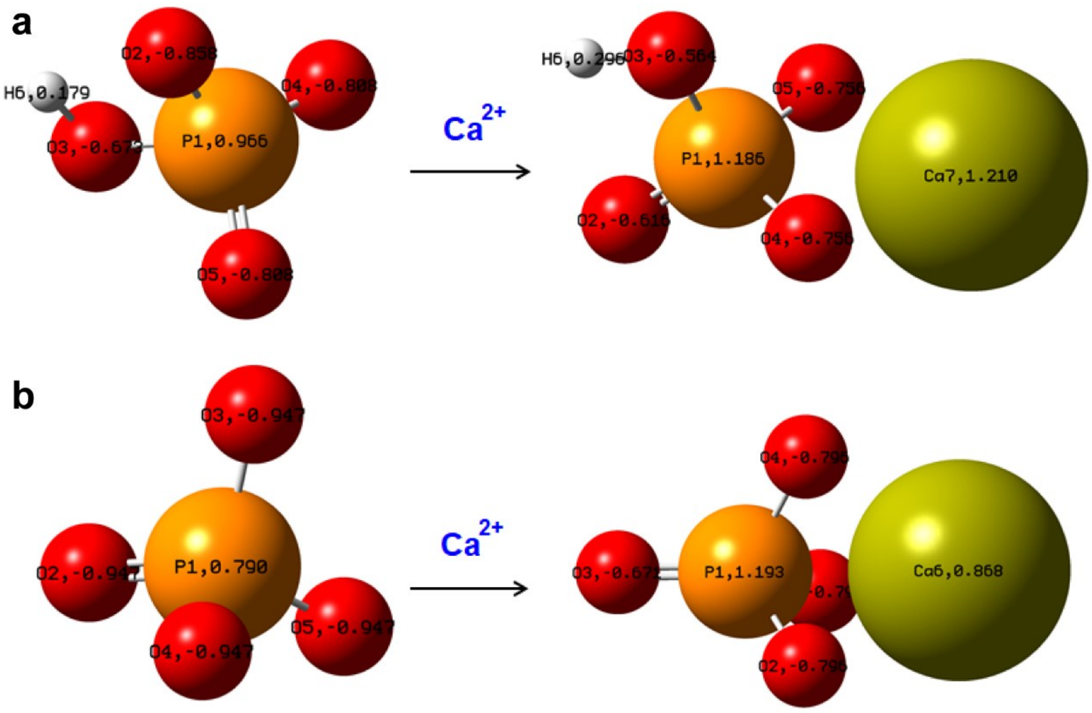

Fig. S1 Optimized atomic structure showing the electrostatic density of (**a**) HPO_4^{2-} and $HPO_4^{2-} + Ca^{2+}$, (**b**) PO_4^{3-} and $PO_4^{3-} + Ca^{2+}$

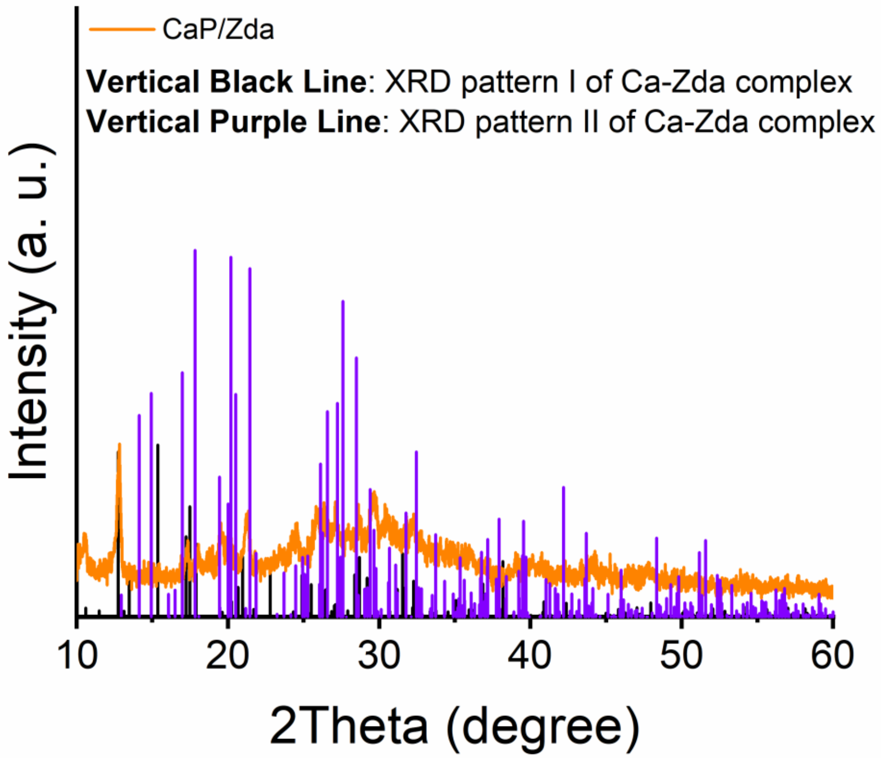

Fig. S2 XRD patterns of CaP/Zda and CaP-Zda complex extracted from cif data provided by reference(Freire et al., 2010) via diamond software, indicating that

CaP/Zda is a complex of calcium and Zda.

Note: 2Theta of CaP/Zda moved 0.3 degrees to the left to calibrate instrumental differences.

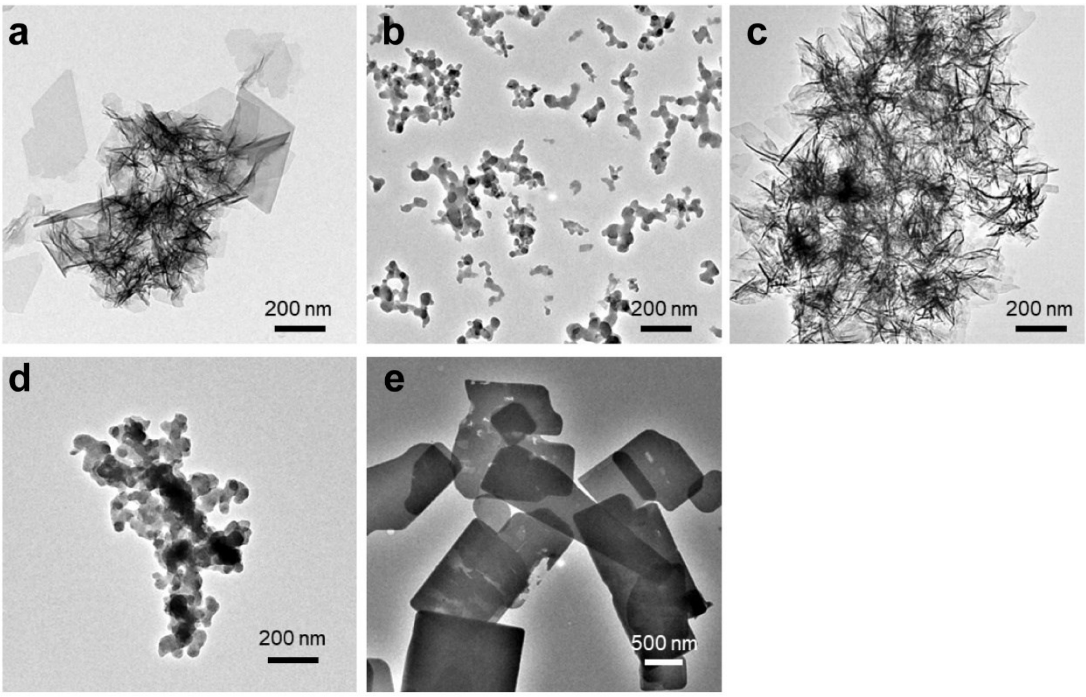

Fig. S3 TEM micrographs of (a) CaP Control-0, (b) CaP/Adn-0, (c) CaP/Cpp-0, (d) CaP/Zda-0 and (e) CaP/Fss-0;

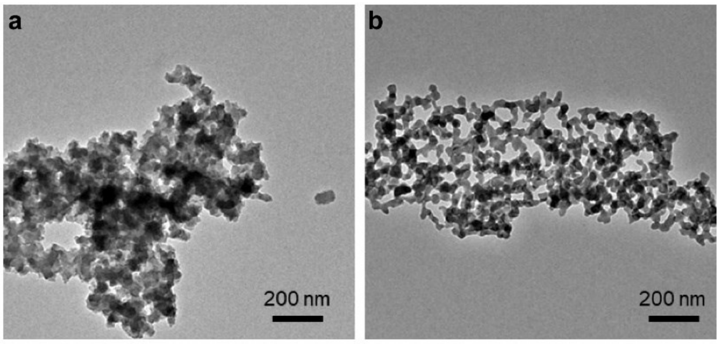

Fig. S4 TEM micrographs of (a) CaP/Adn-1 and (b) CaP/Zda-1;

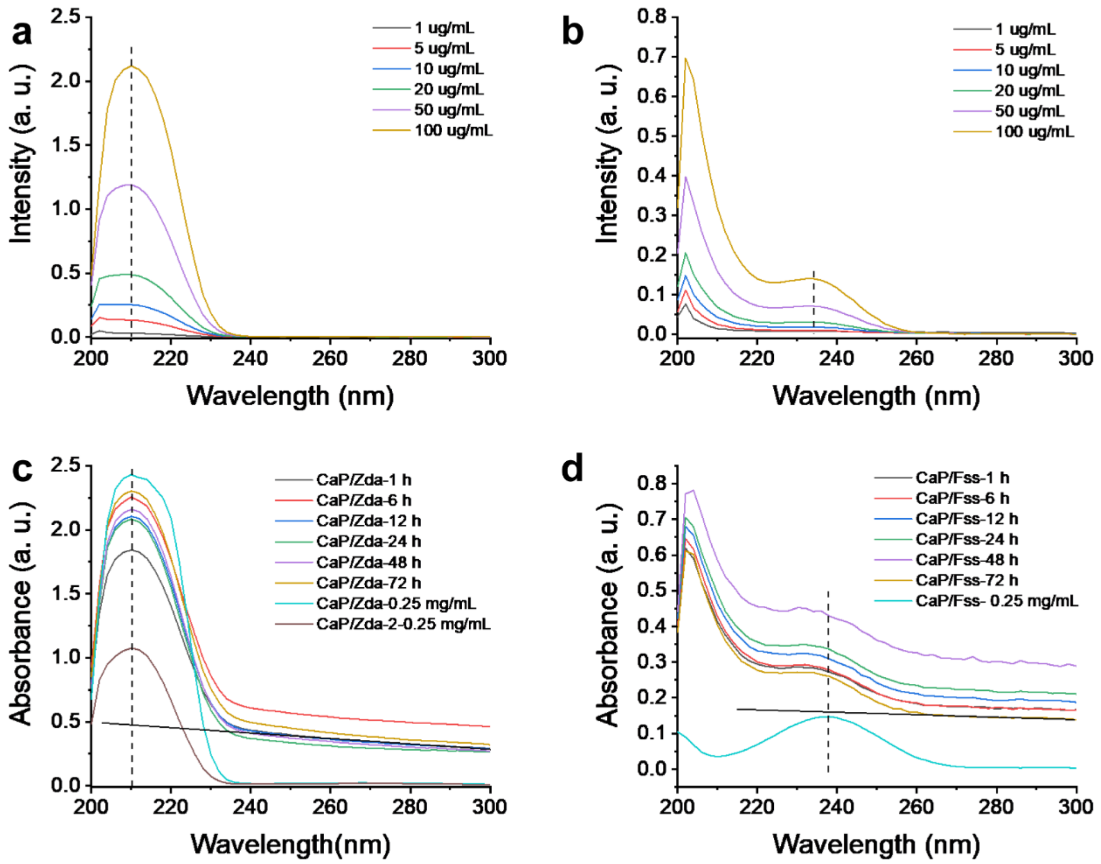

Fig. S5 UV-Vis curves of (a) Zda and (b) Fss in PBS solution with a series of concentration of 1, 5, 10, 20, 50 and 100 μg/mL; UV-Vis curves of released or dissolved (c) Zda in CaP/Zda system and (d) Fss in CaP/Fss system, the black oblique line was subtracted to obtain accurate peak intensity. Every sample was measured for three times, and only one curve was shown here.

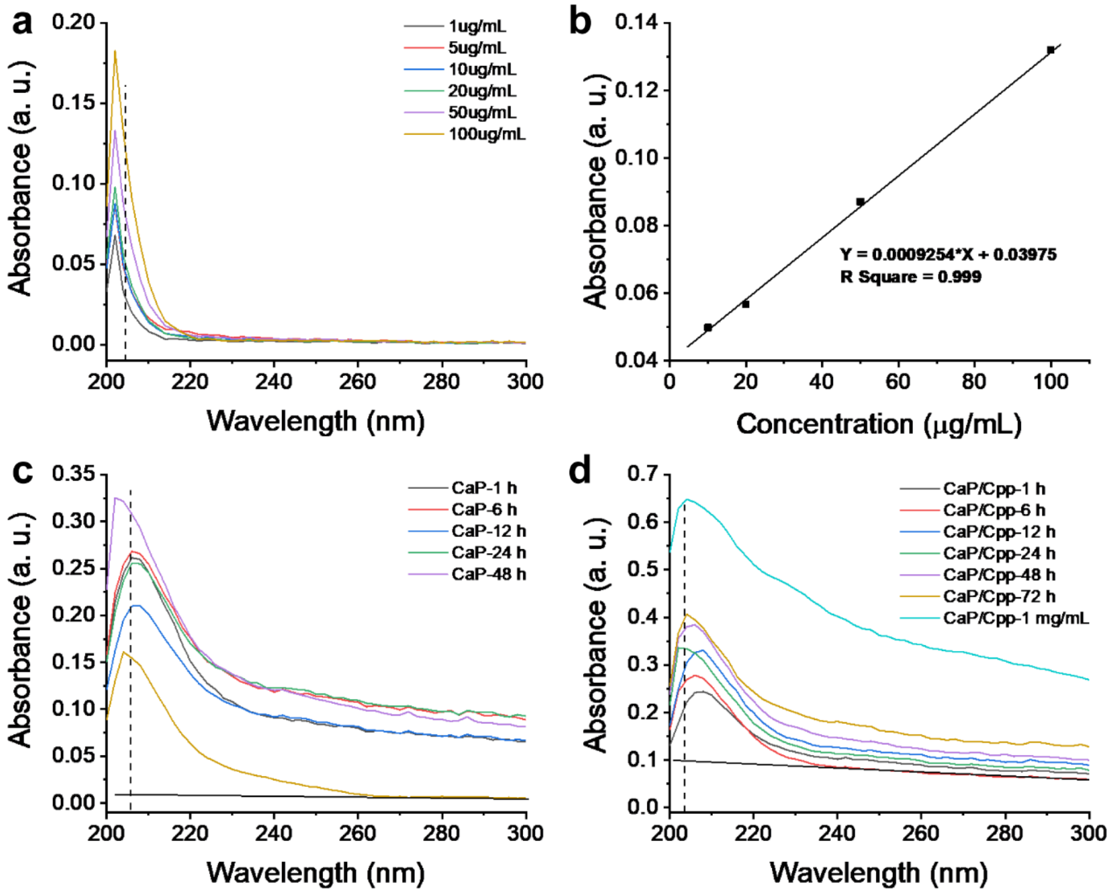

Fig. S6 (a) UV-Vis curves of Cpp in PBS solution with a series of concentration of 1, 5, 10, 20, 50 and 100 μg/mL and (b) related absorbance-concentration curves of Cpp; UV-Vis curves of released solution of (c) CaP, and (d) dissolved Cpp in CaP/Cpp system, the black oblique line was subtracted to obtain accurate peak intensity. Every sample was measured for three times, and only one curve was shown here.
